# Expression Analysis of Five Different Long Non-Coding Ribonucleic Acids in Nonsmall-Cell Lung Carcinoma Tumor and Tumor-Derived Exosomes

**DOI:** 10.3390/diagnostics12123209

**Published:** 2022-12-17

**Authors:** Samaneh Talebi, Asal Jalal Abadi, Golnesa Kazemioula, Nayyerehalsadat Hosseini, Forough Taheri, Saba Pourali, Touba Mahdloo, Marzieh Rezaei, Mohammadreza Mirinezhad, Naser Ajami, Arash Salmaninejad

**Affiliations:** 1 Department of Medical Genetics, Faculty of Medicine, Mashhad University of Medical Sciences, Mashhad 9177899191, Iran; 2 Department of Genetics, Faculty of Advanced Science and Technology, Tehran Medical Sciences, Islamic Azad University, Tehran 1916893813, Iran; 3 Department of Medical Genetics, School of Medicine, Tehran University of Medical Sciences, Tehran 917852689536, Iran; 4 Department of Genetics and Molecular Biology, School of Medicine, Isfahan University of Medical Science, Isfahan 9177856225, Iran; 5 Department of Science, Kish International Campus, University of Tehran, Kish 9177856228, Iran; 6 Department of Medical Biotechnology & Nanotechnology, Faculty of Medicine, Mashhad University of Medical Sciences, Mashhad 9177899191, Iran; 7 Centre de Recherche du CHU de Québec-Université Laval, Quebec City, QC G1A 0A2, Canada; 8 Regenerative Medicine, Organ Procurement and Transplantation Multi-Disciplinary Center, Razi Hospital, School of Medicine, Guilan University of Medical Sciences, Rasht 4183753689, Iran; 9 Department of Medical Genetics, Faculty of Medicine, Tabriz University of Medical Sciences, Tabriz 5164784755, Iran

**Keywords:** nonsmall-cell lung carcinoma, exosomes, long non-coding ribonucleic acids, expression

## Abstract

Long non-coding ribonucleic acids (LncRNAs) are recently known for their role in regulating gene expression and the development of cancer. Controversial results indicate a correlation between the tissue expression of LncRNA and LncRNA content of extracellular vesicles. The present study aimed to evaluate the expression of different LncRNAs in non-small cell lung cancer (NSCLC) patients in tumor tissue, adjacent non-cancerous tissue (ANCT), and exosome-mediated lncRNA. Tumor and ANCT, as well as serum samples of 168 patient with NSCLC, were collected. The GHSROS, HNF1A-AS1, HOTAIR, HMlincRNA717, and LINCRNA-p21 relative expressions in tumor tissue, ANCT, and serum exosomes were evaluated in NSCLC patients. Among 168 NSCLC samples, the expressions of GHSROS (REx = 3.64, *p* = 0.028), HNF1A-AS1 (REx = 2.97, *p* = 0.041), and HOTAIR (REx = 2.9, *p* = 0.0389) were upregulated, and the expressions of HMlincRNA717 (REx = −4.56, *p* = 0.0012) and LINCRNA-p21 (REx = −5.14, *p* = 0.00334) were downregulated in tumor tissue in contrast to ANCT. Moreover, similar statistical differences were seen in the exosome-derived RNA of tumor tissues in contrast to ANCT samples. A panel of the five lncRNAs demonstrated that the area under the curve (AUC) for exosome and tumor was 0.937 (standard error: 0.012, *p* value < 0.0001). LncRNAs GHSROS, HNF1A-AS1, and HOTAIR showed high expression in tumor tissue and exosome content in NSCLC, and a panel that consisted of all five lncRNAs improved diagnosis of NSCLC.

## 1. Introduction

The recent global report on cancer introduced lung cancer as the leading cause of cancer-related morbidity and mortality among men, whereas, in women, it ranks third for incidence, after breast and colorectal cancer, and second for mortality, after breast cancer [1]. By 2020, there were 2,206,771 new cases and 1,796,144 deaths because of lung cancer, accounting for 11.4% of new cases and 18% of new deaths from 36 cancers of all sites [2]. Lung cancer is directly related to tobacco smoking, and it has been estimated that regarding the widespread use of tobacco smoking in both genders, we are facing an increased risk of lung cancer for the next decades [1]. The 5-year survival of lung cancer is reported to be up to 30 percent in different countries, mainly depending on their management strategies and early detection programs [2,3,4]. It has been demonstrated that the early detection of lung cancer improves clinical outcomes and longer survival [2,3,4].

Recent studies mainly focused on identifying novel biomarkers for lung cancer, as the screening programs using computed tomography and lung biopsy are invasive and not cost-effective [5,6]. Among these biomarkers, extracellular vesicles are of great interest. These small particles circulating in the human bloodstream travel to every part of the body. Each extracellular vesicle contains specific proteins or nucleic acid cargos based on the originating tissue. Therefore, these small vesicles may act as messengers or indicators of specific events in their originating tissue [7,8]. Cancerous cells produce extracellular vesicles similar to normal tissue; however, the content of these vesicles is different from the tissue and has diagnostic potentials. Moreover, it has been suggested that tumor-derived extracellular vesicles have the potential to promote tumor progression and metastasis as well as promoting chemoresistance [9,10]. Long non-coding ribonucleic acids (lncRNAs) are among the extracellular vesicles’ cargos. It has been reported that every cancerous tissue has specific lncRNAs, and the extracellular vesicles originating from these tissues may contain different lncRNAs [11,12,13,14]. The extracellular vesicles containing such lncRNAs have the potential to re-program the target cells and build-up a favorable tumor environment facilitating tumor growth and metastasis [10,11,15,16]. Although the role of most of these extracellular vesicles including lncRNAs is addressed in the literature, a different expression of miRNAs between the tumor tissue, extracellular vesicles, and adjacent non-cancerous tissue (ANCT) is not widely studied in non-small cell lung cancer (NSCLC) [17]. Among various lncRNAs introduced to be involved in the development of lung cancer, GHSROS, HMlincRNA717, HNF1A-AS1, HOTAIR, and LNCRNA-p21 are reported to have oncogenic roles [18]. GHSROS overexpression has been linked to increased cancer cell migration in lung cancer patients. Similarly, HMlincRNA717 is reported to play its oncogenic role in NSCLC by mediating metastasis. HNF1A-AS1 and HOTAIR are regulators of the tumor cell cycle and progression in lung cancer [18,19]. Regarding the role of these lncRNAs in the development and progression of lung cancer, and due to a lack of experimental evidence about the effect of these lncRNAs as cargos of extracellular vesicles, the present study aimed to evaluate the differential expression of 5 lncRNAs including GHSROS, HMlincRNA717, HNF1A-AS1, HOTAIR, and LINCRNA-p21 in the tumors, tumor-derived exosomes, and ANCT in NSCLC patients.

## 2. Materials and Methods

### 2.1. Patient Samples

Ethic committees of the Mashhad University of Medical Sciences (1398.765.8) approved the present study, and informed consent was signed by all patients or their families. All patients who had a confirmed diagnosis of NSCLC and were a candidate for surgery enrolled in the present study after giving written informed consent. NSCLC lung cancer subtypes were diagnosed according to the 8th lung cancer TNM classification and clinical staging system. Patients had not received radiotherapy or chemotherapy treatment before the surgery. The cancerous tissue samples and the corresponding ANCTs were excised during surgery from 168 patients admitted to Khorasan Razavi Provincial Omid Hospital with a definite diagnosis of NSCLC. The clinical data of the study population are presented in Table 1. For each patient, venous blood samples were collected in EDTA anticoagulation tubes, and the plasma was separated by centrifugation at 2800× *g* for 5 min and stored at 4 °C. The clear supernatant stored at −80 °C and isolation of plasma EVs, as well as other laboratory methods, were performed based on the Li et al. study as summarized below [20].

### 2.2. GEO Analysis

To predict lncRNAs with significant differential expression profiles in lung cancer, we searched the gene expression omnibus (GEO) database for lung cancers in humans based on the GEO2R analysis and selected GSE160769 and GSE130779 [21]. Considering the expression levels of lncRNAs in the cancerous tissue, ANCT, and exosomes in the patients, their roles in cancer GHSROS, HMlincRNA717, HNF1A-AS1, HOTAIR, and LINCRNA-p21 were selected.

### 2.3. Extracellular Vesicle (EV) Isolation from Plasma

The EV isolation from plasma and analysis were performed based on the Chuang et al. study [20]. According to the manufacturer’s guide, EVs were isolated from plasma using exoRNeasy Serum/Plasma Midi Kit (QIAGEN, Germantown, MD, USA). Samples were centrifuged for 10 min at 16,000× *g* and 4 °C to remove residual cells and debris. The supernatant was transferred into a new tube and mixed adequately with isolation reagents. The supernatant was removed, and the EV pellet was recovered by re-suspending in QIAzol.

### 2.4. Measuring Particle Size of Isolated EVs

The pellets were diluted in PBS and analyzed using the Nanosight NS300 system (Malvern Instruments, Malvern, UK). The distribution of the particle size was measured using Nanosight Tracking Analysis software. Size distribution profiles were averaged across three replicates of each sample to derive the representative size distribution profiles.

### 2.5. Flow Cytometry Analysis of Exosome Marker Proteins

The flow cytometry analysis was used to validate EVs with the general exosome markers including CD81 and CD63. Isolated EV pellets were diluted in PBS, and incubated with FITC-(catalog: ab102884, Abcam, USA)-conjugated anti-CD63 (catalog: ab59479, Abcam, US) or anti-CD81 antibodies (catalog: ab79559, Abcam, US) diluted in PBS/0.5% BSA for 60 min at 37 °C. The EV samples were assayed with a Cytoflex flow cytometer (Beckman, USA), and IgG incubated samples were used as a negative control.

### 2.6. RNA Extraction

Total RNA was isolated from cancerous tissue, ANCTs, and EV samples using the TRIzol™ Reagent (Invitrogen, Carlsbad, CA, USA) and the Direct-zol™ RNA MiniPrep kit (Zymo Research Corp., Irvine, CA, USA) according to the manufacturer’s guidelines. The extracted RNA was treated with DNase-I treatment to remove DNA contamination. The extracted RNA’s quantity and quality were assessed by Nanodrop 2000 Spectrophotometer (Thermo Scientific, Waltham, MA, USA) following gel electrophoresis.

### 2.7. cDNA Synthesis and Quantitative Real-Time Reverse Transcription PCR (qRT-PCR)

cDNA was made using a reverse transcriptase kit (Applied Biosystems High-Capacity cDNA Reverse Transcription, USA). A TaqMan Probe-Based Real-Time PCR Assay was performed, and the HPRT1 gene was considered as the reference gene. Primers and probes used for the PCR were designed using the Allele ID 7 software (Premier Biosoft, Palo Alto, CA, USA) (Table S1). An Applied Biosystems TaqMan^®^ Universal PCR Master Mix was used for quantification of lncRNA expression. Amplifying conditions were 5 min for the denaturation step at 95 °C, followed by 40 cycles of 95 °C for 10 s and 60 °C for 50 s, and a final extension step at 72 °C for 7 min.

### 2.8. Statistical Analysis

The relative expression of lncRNAs in tumor tissues compared with ANCTs, in plasma tumor-derived exosomes, and in tumor tissues compared with exosome samples were determined based on the calculation of Ln [Efficiency^ΔCT^] values. The Chi-square test evaluated the association between lncRNAs’ transcript level and clinical data. Pearson’s correlation coefficient was used to estimate the correlation among expression levels of lncRNAs and between relative expressions of LncRNAs and age. Mean values of the expression of genes in different tissues were compared between subgroups using the *one*-*way analysis* of variance (*ANOVA*) with Bonferroni post hoc. *p* values less than 0.05 were considered significant. A binary logistic regression was used to calculate the predictive probability of combined biomarkers for the ROC analysis. Areas under the curves (AUCs) were used to evaluate the diagnostic value of the combination of lncRNAs.

## 3. Results

### 3.1. Characterization of Plasma Isolated EVs

**Figure 1:**
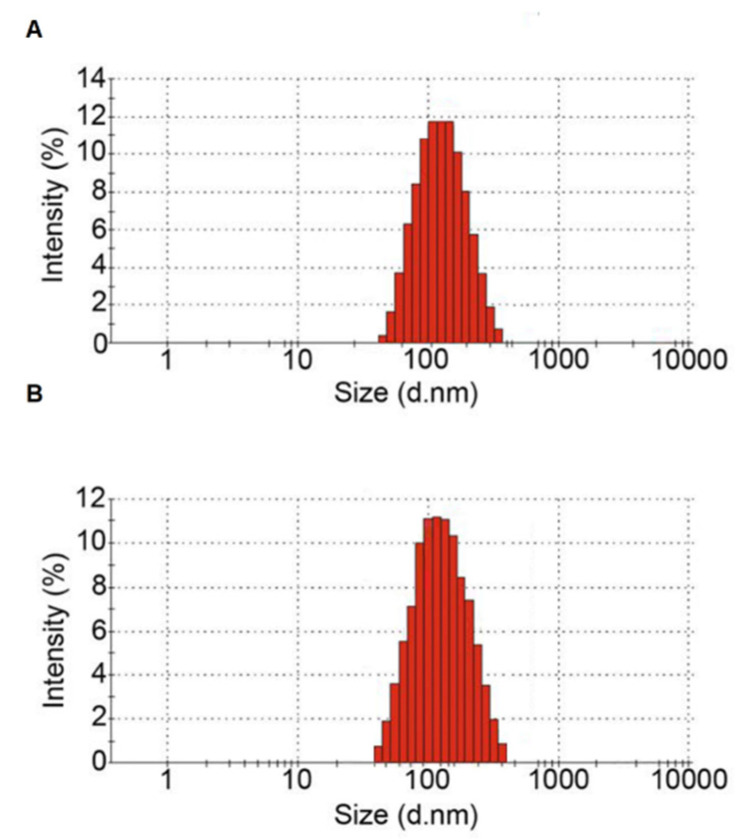
Distribution of extracellular vesicle size in (**A**) ADC samples and (**B**) SQCC samples.

**Figure 2:**
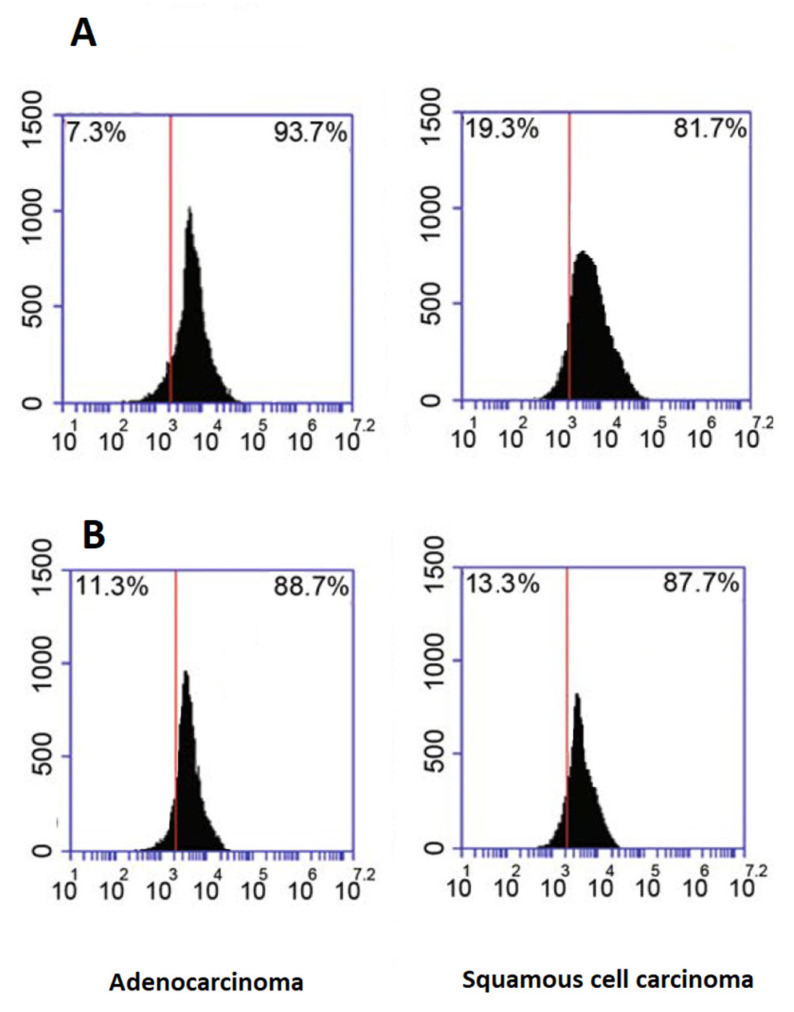
Flow cytometry results for the exosomal markers’ antibodies. Line (**A**): CD81; Line (**B**): CD63.

A nanoparticle tracking analysis and flow cytometry were used to analyze plasma exosomes. The nanoparticle tracking analysis demonstrated that the extracted EVs mostly included small EVs with mainly the desirable size for exosomes, ranging from 100 to 200 nm (Figure 1) (Table 2). The flow cytometry analysis confirmed the presence of CD63 and CD81 as markers of extracellular vesicles (Figure 2).

### 3.2. The Relative Expressions of lncRNAs

**Figure 3:**
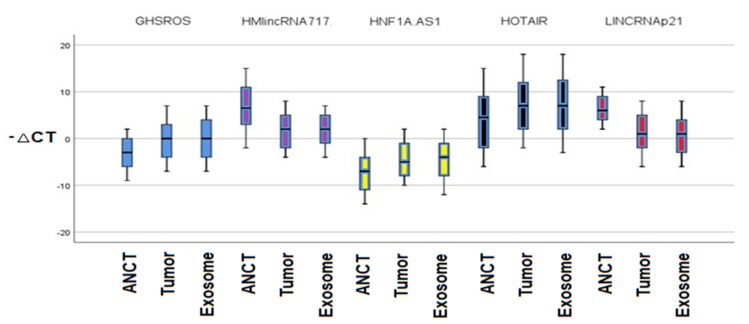
Relative expression of different lncRNAs in different tissues.

There was a significant difference in the relative expression of lncRNAs in the tumor and ANCT tissues (Figure 3). lncRNA-GHSROS, lncRNA-HNF1A-AS1, and lncRNA-HOTAIR were upregulated (*p* < 0.001, relative expression = 3.41071; *p* < 0.001, relative expression = 2.79167, and *p* < 0.001, relative expression = 3.02381, respectively), while the lncRNA-P21 and lncRNA-HMlincRNA717 were downregulated (*p* < 0.001, relative expression = −5.16667 and *p* < 0.001, relative expression = −5.07143) in tumor tissue in comparison to normal tissue. The lncRNAs’ relative expression between tumor tissue and exosomes was not different among the studied lncRNAs. Moreover, the relative expression between lncRNAs in exosome and normal tissue was significantly upregulated in lncRNA-GHSROS, lncRNA-HNF1A-AS1, and lncRNA-HOTAIR (*p* < 0.001, relative expression = 3.32738; *p* < 0.001, relative expression = 2.55357 and *p* < 0.001 relative expression = 3.01190). LncRNA-HMlincRNA717 and lncRNA-P21 were significantly downregulated in exosomes in contrast to the normal tissue (*p* < 0.001, relative expression = −5.06548 and *p* < 0.001, relative expression = −5.58929) (Table 3).

### 3.3. The Correlation between lncRNA Expressions among Different Tissues and the Area under the Curve

**Figure 4:**
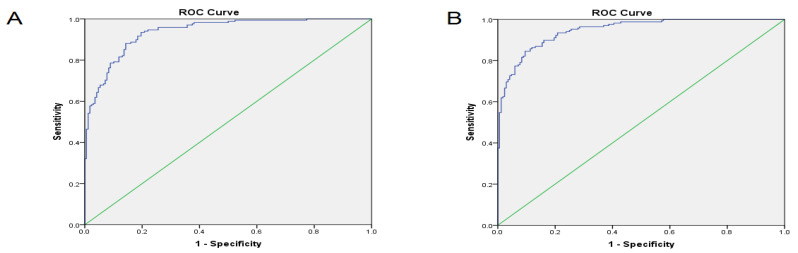
The ROC curve analysis results. (**A**): The area under the curve (AUC) for the expression of a panel of 5 lnc-RNAs in tumor and normal tissue. (**B**): The AUC for the expression of a panel of 5 lnc-RNAs in exosome and normal tissue.

The Pearson correlation test revealed that the expression of lncRNA-GHSROS in ANCT was correlated with lncRNA-P21 of the tumor (*p* < 0.05, C = 0.167), the expression of exosomal lncRNA-GHSROS was correlated with lncRNA-HMlincRNA717 in the tumor tissue (*p* < 0.05, C = 0.178), and lncRNA-GHSROS of the exosome was correlated with exosomal lncRNA-P21 (*p* < 0.05, C = 0.178). lncRNA-HMlincRNA717 of the tumor tissue was correlated with lncRNA-P21 of ANCT (*p* < 0.05, C = 0.157), and lncRNA-HNF1A-AS1 of ANCT was correlated with lncRNA-P21 of ANCT (*p* < 0.001, C = −0.205). The lncRNA-HNF1A-AS1 of the exosome was correlated with lncRNA-HOTAIR of the exosome (*p* < 0.05, C = −0.173). The lncRNA-HNF1A-AS1 of the exosome was correlated with the lncRNA-P21 of the exosome (*p* < 0.05, C = 0.190), and the lncRNA-HOTAIR of the exosome was correlated with lncRNA-HNF1A-AS1 of the exosome (*p* < 0.05, C = −0.173). The lncRNA-P21 of ANCT was correlated with the lncRNA-HMlincRNA717 of the tumor tissue (*p* < 0.05, C = 0.157), and the lncRNA-P21 of ANCT was correlated with lncRNA-P21 of the tumor tissue (*p* < 0.05, C = −0.157). The lncRNA-P21 of the tumoral tissue was correlated with the lncRNA-P21 of ANCT (*p* < 0.05, C = 0.157), and the lncRNA-P21 of the exosome was correlated with lncRNA-GHSROS of the exosome (*p* < 0.05, C = −0.178). The binary logistic regression model results demonstrated that an odds ratio for all five Lnc-RNAs expression was significant (*p* < 0.05) in tumor tissue compared to ANCT and exosome compared to ANCT (Table 4). Considering a panel of the five lncRNAs demonstrated that the AUC for tumor and ANCT was 0.937 (standard error: 0.012, *p* value < 0.0001). These lncRNAs in ANCT and exosome demonstrated an AUC of 0.947 (standard error: 0.011, *p* value < 0.0001) (Figure 4, Table 5). The AUC of 0.947 means that using the combination of these lncRNAs will have excellent discrimination for NSCLC.

### 3.4. The Association between lncRNA Expression and Clinicopathological Data

Considering the expression differences as upregulation or downregulation for different lncRNAs, the lncRNA-GHSROS expression in the tumor tissues and ANCT was correlated with smoking (*p* = 0.005). The lncRNA-HOTAIR expression in the tumor tissues and exosomes was correlated with smoking (*p* = 0.044). The relative expression of the lncRNA-HMlincRNA717, lnc-P21, and lncRNA-HNF1A-AS1 in different tissues was not correlated with any of the clinicopathological data (Table 6).

## 4. Discussion

According to our study results, the relative expressions of 5 different lncRNAs including GHSROS, HMlincRNA717, HNF1A-AS1, HOTAIR, and P21 are different among the NSCLC tissue, ANCT, and plasma exosomes. The lncRNA-GHSROS, lncRNA-HNF1A-AS1, and lncRNA-HOTAIR were upregulated and the lncRNA-P21 and lncRNA-HMlincRNA717 were downregulated in tumor tissue in comparison to normal tissue. The relative expression between lncRNAs in exosome and normal tissue was significantly upregulated for lncRNA-GHSROS, lncRNA-HNF1A-AS1, and lncRNA-HOTAIR and downregulated for exosomal lncRNA-HMlincRNA717 and lncRNA-P21 in contrast to ANCT. The lncRNAs’ relative expression between tumor tissue and exosomes was not different among the studied lncRNAs.

Lung cancer is a leading cause of mortality and morbidity, and early diagnosis has been linked to better overall survival [20,22,23]. Among various screening methods, serum biomarkers are a growing field in the early detection of various cancers including NSCLC. The role of lncRNAs as a regulator of many cellular processes is evident, and therefore the aberrant expression of these RNA molecules has been linked to the development of cancers. Similar to other RNA molecules, lncRNAs are secreted from various cells and also can be found in body fluids [20,24]. The secreted lncRNAs act as a messenger and can be packed in extracellular vesicles including exosomes. Recently, the exosomal lncRNAs in patients with cancers had provided both diagnostic and prognostic clues for the management of their malignancies [20,25]. We demonstrated different expression levels of 5 lncRNAs, which have been thought to have oncogenic or tumor suppressor roles in various cancers in the exosomes, ANCT, and tumor tissues of patients with NSCLC.

The lncRNA-GHSROS is an oncogenic lncRNA upregulated in many cancers. The gene coding lncRNA-GHSROS is antisense located on the ghrelin receptor encoding a 1.1 kb transcript [26]. It has been reported that lncRNA-GHSROS is expressed in prostate and breast cancers, and its overexpression is correlated with advanced tumor stages [25,27]. Overexpression of this lncRNA results in increased cellular proliferation and vascularization of tumor tissues [27]. It has been demonstrated that increased expression of this lncRNA has been linked to the migration of A549 lung cancer cell lines [26]. Our study showed that lncRNA-GHSROS is upregulated in tumor tissue compared to the ANCT, which is in line with the previous research by Whiteside et al. Moreover, we revealed that this lncRNA is significantly overexpressed in plasma exosomes in contrast to the ANCT in NSCLC patients. However, the expression of this lncRNA is not significantly different among tumor tissues and plasma exosomes. Moreover, the expression is not related to the clinical stage or subtype of lung cancer.

The LncRNA-HMlincRNA717 has been widely studied in gastric cancer [28]. The LncRNA-HMlincRNA717 or “gastric cancer-associated transcript-2” is among the 135 lncRNAs with aberrant expression in gastric cancer downregulated in cancer cell lines in contrast to the normal tissue [28]. The expression level of LncRNA-HMlincRNA717 is related to venous invasion and distant metastasis [28]. Like gastric cancer, a decreased level of this lncRNA is associated with the poor overall survival of patients with pancreatic cancer [29]. Similarly, downregulation of LncRNA-HMlincRNA717 has been related to the poor prognosis of patients with lung cancer [30]. A study by Xie et al. reported that the expression of LncRNA-HMlincRNA717 in NSCLC is related to metastasis and invasion, and this lncRNA can be used as a prognostic marker for lung tumors [30]. Similarly, we demonstrated that this LncRNA-HMlincRNA717 is downregulated in lung cancer tissue in contrast to the ANCT. Furthermore, we demonstrated that this lncRNA expression level has been downregulated in plasma exosomes compared to the ANCT. However, there is no significant relationship between the expression level of this lncRNA and tumor stage or subtype.

HOTAIR is a 2.1 kb nucleotide non-coding RNA and a transcript from HOXC locus [31]. This lncRNA interacts with various genes including HOXA5 and MMP. The lncRNA-HOTAIR promotes exosome secretion from hepatocellular carcinoma and is a novel biomarker introduced for various types of cancer. The level of this circulating lncRNA has been considered as a diagnostic marker in breast cancer patients. LncRNA-HOTAIR enhances tumor proliferation and migration in many cancers including gastric cancer, hepatocellular carcinoma, colorectal cancer, and prostate cancer [32]. To date, only a few studies evaluated the role of exosomal lncRNA-HOTAIR in cancer. The overexpression of exosomal lncRNA-HOTAIR has been described as an indicator of poor prognosis, and also as a salivary biomarker for the early diagnosis of pancreatic cancer [33]. Wang et al. reported that exosomal HOTAIR is positively correlated with the HER2/neu status of breast cancer [34]. Zhang et al. study showed that exosomal HOTAIR promotes the invasion and proliferation of lung cancer [35]. Similarly, according to our results, the expression level of lncRNA-HOTAIR is greater in tumoral tissue in contrast to the ANCT, and plasma exosomes in comparison to the ANCT.

lncRNA-P21 is a tumor suppressor which is mainly linked to p53 activity. lncRNA-p21 has been reduced in many cancers including hepatocellular carcinoma, prostate cancer, and gastric cancer. In NSCLC, the lncRNA-p21 is also downregulated, and it has been demonstrated that tumor cells with overexpression of lncRNA-p21 have a higher microvascular density and decreased expression of E-cadherin [36]. Ao et al. demonstrated that lncRNA-p21 can inhibit NCSLC proliferation and migration [37]. Castellano et al. demonstrated that the expression of lncRNA-p21 is related to the prognosis in lung cancer and regulation of angiogenesis [36]. Moreover, this lncRNA can be found in body fluids, including blood, urine and plasma, and exosomes [8]. A study by Işın et al. demonstrated lncRNA-p21 is present in urine exosomes of prostate cancer patients [38]. Moreover, the exosomal level of lncRNA-p21 is a useful marker in distinguishing prostate cancer from benign prostate disease [38]. Our results demonstrated that lncRNA-p21 has decreased tumor tissue and serum exosome expression in contrast to the ANCT in NSCLC patients. A study by Gezer et al. demonstrated that HOTAIR and lincRNA-p21, which show relatively low expression in Hela and MCF-7 cell lines, are enriched in exosomes [39]. After inducing DNA damage, the lncRNA-p21 is a reliable predictor of exposure to bleomycin in contrast to the other lncRNAs [39].

We demonstrated that the lncRNA-HNF1A-AS1 has increased relative expression in tumoral tissue and plasma exosome in contrast to ANCT. The Liu et al. study showed that lncRNA-HNF1A-AS1 upregulates in lung cancer tissue and cell lines [40]. They demonstrated that the overexpression of lncRNA-HNF1A-AS1 is linked to NSCLC proliferation and invasion [40]. Ma et al. reported that overexpression of this lncRNA is related to poor prognosis in NSCLC patients [41]. However, the exosomal lncRNA-HNF1A-AS1 has not been widely studied in the literature. Luo et al. reported that exosomal lncRNA-HNF1A-AS1 secreted by the cisplatin-resistant cells could inhibit apoptosis of cervical tumors [42,43]. Moreover, the inhibition of exosomal lncRNA-HNF1A-AS1 could inhibit tumor formation in nude mice [42].

Considering a panel of these lncRNAs may be helpful in the determination of other malignancies, and further studies may consider more lung cancer-specific lncRNAs in combination with the five lncRNAs to consider a higher diagnostic accuracy for the detection of NSCLC. Furthermore, the stage of NSCLC may affect the helpfulness of considering these five lncRNAs as a diagnostic marker while we consider NSCLC from every stage. Further studies may consider specific NSCLC stages to achieve results that are more reliable.

One of the major limitations of our study was the limited sample size and absence of a control group. We used ANCT as a normal tissue, but further studies may consider normal tissues besides ANCT. Moreover, the five lncRNAs including GHSROS, HNF1A-AS1, HOTAIR, HMlincRNA717, and LINCRNA-p21 are not specific for NSCLC.

## 5. Conclusions

The present study demonstrated that the lncRNA-GHSROS, lncRNA-HNF1A-AS1, lncRNA-HOTAIR, lncRNA-P21, and lncRNA-HMlincRNA717 are aberrantly regulated in NSCLC. The relative expression between lncRNAs in exosome and normal tissue was significantly upregulated for lncRNA-GHSROS, lncRNA-HNF1A-AS1, and lncRNA-HOTAIR, and downregulated for exosomal lncRNA-HMlincRNA717 and lncRNA-P21 in contrast to ANCT. In addition, the lncRNA relative expression between the tumor tissue and exosomes was not different among the studied lncRNAs. None of the 5 lncRNAs were correlated with tumor stage, tumor subtype, body mass index, age, and gender. Only the lncRNA-HOTAIR expressions in the tumor tissues and exosomes were correlated with smoking. According to our findings, considering a panel of five lncRNAs including lncRNA-GHSROS, lncRNA-HNF1A-AS1, lncRNA-HOTAIR, lncRNA-P21, and lncRNA-HMlincRNA717 can be helpful in the diagnosis of NSCLC.

## Figures and Tables

**Table 1:** The frequency of demographic data of study population.

Variable		Number
Gender N (%)	Female	54 (32.1)
	Male	114 (67.9)
Mean age ± SD		62.22 ± 6.59
Subtype N (%)	Adenocarcinoma	116(69)
	Squamous cell carcinoma	52 (31)
Stage N (%)	1	47 (28)
	2	71 (42.3)
	3	50 (29.8)
Body mass index N (%)	<30	110 (65.5)
	>30	58 (34.5)
Smoking		147 (87.5)

**Table 2:** Characterization of EV particle diameter.

Sample	ADC	SQCC
Average of particle diameter (nm)	121.2	118.6
Polydispersity index (PDI) ^a^	0.247	0.231
Major peak of particle diameter (nm)	171.4	159.8
Percentage of 20–200 nm diameter (%)	81.4	79.8

ADC: adenocarcinoma; SQCC: squamous cell carcinoma. ^a^ Polydispersity index (PDI) is a dimensionless value that represents the distribution of particle size. PDI values of 0.08–0.7 indicate moderate dispersion system and optimum application scope of algorithm.

**Table 3:** Comparison of relative expression of lncRNAs in different tissues.

Genes	Target Sample	Compared To	Relative Expression	Std. Error	*p* Value	95% Confidence Interval	
						Lower Bound	Upper Bound
GHSROS	tumor	normal	3.41071 *	0.43620	0.000	2.3629	4.4585
		exosome	0.08333	0.43620	1.000	−0.9644	1.1311
	exosome	normal	3.32738 *	0.43620	0.000	2.2796	4.3752
		tumor	−0.08333	0.43620	1.000	−1.1311	0.9644
HMlincRNA717	tumor	normal	−5.07143 *	0.43958	0.000	−6.1273	−4.0155
		exosome	−0.00595	0.43958	1.000	−1.0618	1.0499
	exosome	normal	−5.06548 *	0.43958	0.000	−6.1214	−4.0096
		tumor	0.00595	0.43958	1.000	−1.0499	1.0618
HNF1A-AS1	tumor	normal	2.79167 *	0.44683	0.000	1.7184	3.8650
		exosome	0.23810	0.44683	1.000	−0.8352	1.3114
	exosome	normal	2.55357 *	0.44683	0.000	1.4803	3.6269
		tumor	−0.23810	0.44683	1.000	−1.3114	0.8352
HOTAIR	tumor	normal	3.02381 *	0.68063	0.000	1.3889	4.6587
		exosome	0.01190	0.68063	1.000	−1.6230	1.6468
	exosome	normal	3.01190 *	0.68063	0.000	1.3770	4.6468
		tumor	−0.01190	0.68063	1.000	−1.6468	1.6230
LINCRNA-p21	tumor	normal	−5.16667 *	0.42132	0.000	−6.1787	−4.1546
		exosome	0.42262	0.42132	0.949	−0.5894	1.4347
	exosome	normal	−5.58929 *	0.42132	0.000	−6.6013	−4.5772
		tumor	−0.42262	0.42132	0.949	−1.4347	0.5894

* The mean difference is significant at the 0.05 level.

**Table 4:** Binary regression model results.

	Tumor vs. ANCT			Exosome vs. ANCT		
Gene	Standard Error	Odds Ratio	*p* Value	Standard Error	Odds Ratio	*p* Value
GHSROS	0.050	1.308	0.000	0.048	1.234	0.000
HMlincRNA717	0.045	0.771	0.000	0.048	0.759	0.000
HNF1A-AS1	0.045	1.151	0.000	0.045	1.169	0.000
HOTAIR	0.029	1.085	0.000	0.029	1.101	0.000
LINCRNA-p21	0.059	0.689	0.000	0.055	0.672	0.000

**Table 5:** The results of ROC curve analysis.

Specificity (%)	Sensitivity (%)	Negative Predictive Value (%)	Positive Predictive Value (%)	AUC (%)	Cut off Point	Gene	
76.4	71.5	68.4	76.1	73.4	>3.51	GHSROS	Tumor compared to ANCT
71.3	72.3	70.1	70.4	67.7	>2.59	HNF1A-AS1	
89.8	91.6	87.7	91.1	85.4	>2.71	HOTAIR	
75.6	76.6	77.6	84.3	73.9	>−4.42	HMlincRNA717	
82	81.4	80.4	79.2	68.1	>−5.02	LINCRNA-p21	
94.4	93.7	91.3	93.8	93.7		Combined LncRNAs	
77.3	74.1	71.1	76.2	75.1	>3.12	GHSROS	Exosome compared to ANCT
70.8	73.4	70.2	69.8	68.1	>2.09	HNF1A-AS1	
88.6	92.2	88.4	92.1	87.2	>1.87	HOTAIR	
74.7	78.7	75.6	86.7	72.2	>−4.86	HMlincRNA717	
83.4	82.1	81.3	76.4	68.5	>−6.67	LINCRNA-p21	
95.7	94.2	94.1	93.6	94.7		Combined LncRNAs	

**Table 6:** The relationship between 5 lncRNAs and clinicopathological data.

**GHSROS**									
	**Tumoral: ANCT (N)**			**Tumoral: Exosome (N)**			**Exosome: ANCT (N)**		
	**Down**	**Up**	***p* Value**	**Down**	**Up**	***p* Value**	**Down**	**Up**	***p* Value**
Age									
<60 years	15	45	0.893	27	33	0.612	15	45	0.794
>60 years	26	82		53	55		29	79	
Gender									
Female	12	42	0.65	25	29	0.813	16	38	0.485
Male	29	85		55	59		28	86	
Subtype									
SCC	13	39	0.904	22	30	0.356	13	39	0.814
Adeno	28	88		58	58		31	85	
Stage									
1	14	33		20	27		18	29	
2	15	56	0.561	33	38	0.513	14	57	0.073
3	12	38		27	23		12	38	
Smoking									
Yes	40	107	0.005	75	72	0.599	75	72	0.484
No	14	7		12	9		9	12	
BMI									
<30	28	82	0.663	56	54	0.24	30	80	0.66
>30	13	45		24	34		14	44	
**HMlincRNA717**									
	** Tumoral: ANCT (N) **			** Tumoral: Exosome (N) **			** Exosome: ANCT (N) **		
	** Down **	** Up **	*** p *** ** Value **	** Down **	** Up **	*** p *** ** Value **	** Down **	** Up **	*** p *** ** Value **
Age									
<60 years	46	14	0.978	29	31	0.258	50	10	0.657
>60 years	83	25		62	46		87	21	
Gender									
Female	45	9	0.167	33	21	0.214	61	13	0.196
Male	84	30		58	56		96	18	
Subtype									
SCC	35	17	0.051	23	29	0.084	43	9	0.798
Adeno	94	22		68	48		94	22	
Stage									
1	38	9		25	22		39	8	
2	56	15	0.387	42	29	0.473	62	9	0.097
3	35	15		24	26		36	14	
Smoking									
Yes	64	83	0.637	73	74	0.861	70	77	0.102
No	8	13		10	11		14	7	
BMI									
<30	85	25	0.837	30	50	0.892	87	23	0.258
>30	44	14		31	27		50	8	
**HNF1A-AS1**									
	** Tumoral: ANCT (N) **			** Tumoral: Exosome (N) **			** Exosome: ANCT (N) **		
	** Down **	** Up **	*** p *** ** Value **	** Down **	** Up **	*** p *** ** Value **	** Down **	** Up **	*** p *** ** Value **
Age									
<60 years	23	37	0.07	26	34	0.981	22	38	0.943
>60 years	27	81		47	61		39	69	
Gender									
Female	12	42	0.141	22	32	0.626	16	28	0.215
Male	38	76		51	63		45	69	
Subtype									
SCC	13	39	0.366	20	32	0.382	16	36	0.317
Adeno	37	79		53	63		45	71	
Stage									
1	16	31		14	33		19	28	0.764
2	16	55	0.21	32	39	0.052	24	47	
3	18	32		27	23		18	32	
Smoking									
Yes	70	77	0.1	61	86	0.475	66	81	0.067
No	6	15		7	14		5	16	
BMI									
<30	31	79	0.537	47	63	0.794	38	72	0.513
>30	19	39		26	32		23	35	
**HOTAIR**									
	** Tumoral: ANCT (N) **			** Tumoral: Exosome (N) **			** Exosome: ANCT (N) **		
	** Down **	** Up **	*** p *** ** Value **	** Down **	** Up **	*** p *** ** Value **	** Down **	** Up **	*** p *** ** Value **
Age									
<60 years	27	33	0.105	29	31	0.473	21	39	0.827
>60 years	35	73		46	62		36	72	
Gender									
Female	21	33	0.714	25	29	0.767	21	33	0.35
Male	41	73		50	64		36	78	
Subtype									
SCC	24	28	0.096	24	28	0.792	21	31	0.237
Adeno	38	78		51	65		36	80	
Stage									
1	17	30		23	24		16	31	
2	32	39	0.1	31	40	0.771	29	42	0.156
3	13	37		21	29		12	38	
Smoking									
Yes	58	89	0.475	57	90	0.044	62	85	0.234
No	10	11		13	8		6	15	
BMI									
<30	38	72	0.383	48	62	0.718	36	74	0.651
>30	24	34		27	31		21	37	
**LINCRNA-p21**									
	** Tumoral: ANCT (N) **			** Tumoral: Exosome (N) **			** Exosome: ANCT (N) **		
	** Down **	** Up **	*** p *** ** Value **	** Down **	** Up **	*** p *** ** Value **	** Down **	** Up **	*** p *** ** Value **
Age									
<60 years	52	8	0.808	29	31	0.369	53	7	0.57
>60 years	95	13		60	48		92	16	
Gender									
Female	45	9	0.261	27	27	0.595	47	7	0.85
Male	102	12		62	52		98	16	
Subtype									
SCC	42	10	0.077	25	27	0.394	44	8	0.669
Adeno	105	11		64	52		101	15	
Stage									
1	40	7		24	23		38	9	
2	60	11	0.252	37	34	0.872	63	8	0.436
3	47	3		28	22		44	6	
Smoking									
Yes	90	57	0.63	92	55	0.223	89	58	0.765
No	14	7		16	5		12	9	
BMI									
<30	94	16	0.27	57	53	0.679	94	16	0.657
>30	53	5		32	26		51	7	

## Data Availability

Data will be available by requesting from correspondence.

## Supplementary Materials

The following supporting information can be downloaded at: https://www.mdpi.com/article/10.3390/diagnostics12123209/s1, Table S1: The sequences of primers for 5 lncRNAs.
