# Supplementary material for: Expression Analysis of Five Different Long Non-Coding Ribonucleic Acids in Nonsmall-Cell Lung Carcinoma Tumor and Tumor-Derived Exosomes
Source: Diagnostics (Basel). 2022 Dec 17;12(12):3209. doi: 10.3390/diagnostics12123209 (PMC9777400; doi:10.3390/diagnostics12123209)
Supplement: Supplementary file 1 [file diagnostics-12-03209-s001.zip › diagnostics-2022170-SI.pdf]

Table **S1**. The sequences of primers for 5 lncRNAs.

Gene Name	Primer & Probe Sequence	length	Product length
GHSROS	F: TGTCGTGGGTGCCTCGCT	18	116
	R: ATAAATATCGCCCTACGTGG	20	
	FAM-AAGCAAACCGTGAAAATGCTGG- TAMRA	22	
HMlincRNA717	F: TGGATGCTTACAAAGGACTGG	21	173
	R: CTGCAATTACGGAAAGAGCTG	21	
	FAM- CGTTGCTCCTCAACCCGG- TAMRA	19	
HNF1A-AS1	F: CAAGAAATGGTGGCTATGA	19	184
	R: TGGACTGAAGGACAAGGGT	19	
	FAM- TCAGAGCTCTTTGGCAATTC- TAMRA	20	
HOTAIR	F: ACCTCTGTCTGTGAGTGCC	19	128
	R: AGAGGAGGGAAGAGAGCGCC	20	
	FAM- ACCCAAGCTAGAGTGCAGTGGCCT- TAMRA	24	
LINCRNA-p21	F: CCCGGGCTTGTCTTTTGTT	19	160
	R: GAGTGGGTGGCTCACTCTTCTG	22	
	FAM- ATGCGGCCTTGCAGG-TAMRA	15	
HPRT1	F: AGCCTAAGATGAGAGTTC	18	88
	R: CACAGAACTAGAACATTGATA	21	
	FAM- CATCTGGAGTCCTATTGACATCGC -TAMRA	24	
